# (In)Sensitivity to Accuracy? Children’s and Adults’ Decisions About Who to Trust: The Teacher or the Internet

**DOI:** 10.3389/fpsyg.2020.551131

**Published:** 2020-08-28

**Authors:** Silvia Guerrero, Carla Sebastián-Enesco, Irene Morales, Elena Varea, Ileana Enesco

**Affiliations:** ^1^Departamento de Psicología, Facultad de Educación, Universidad de Castilla La Mancha, Toledo, Spain; ^2^Sección Departamental de Investigación y Psicología en la Educación, Universidad Complutense de Madrid, Madrid, Spain

**Keywords:** selective trust, accuracy, individual differences, internet, teacher

## Abstract

Recent research has challenged the extended idea that when presented with conflicting information provided by different sources, children, as do adults, make epistemic judgments based on the past accuracy of each source. Instead, individuals may use relatively simple, but adaptive non-epistemic strategies. Here we examined how primary-school children (*N* = 114) and undergraduate students (*N* = 57) deal with conflicting information provided by two key sources of information in their day-to-day lives: their teacher and the Internet. In order to study whether the inaccuracy of a source generated a decline in trust, we manipulated this variable between participants: teacher-wrong and Internet-wrong conditions. For this, we first presented two baseline trials, followed by the accuracy manipulation, and finally, two post-test trials. Analyses were performed on group performance as well as on individual performance, to explore the individual patterns of responses. Results revealed that most participants showed no preference for any source during baseline, with no age differences in their overall choices. Crucially, when a given source provided inaccurate information about a familiar issue, most children and adults did not lose trust on this source. We propose tentative explanations for these findings considering potential differences in the participants’ strategies to approach the task, whether or not epistemic.

## Introduction

Testimony is essential for humans to learn a vast number of concepts and information about our present and past world. In many cases, the testimonies provided by different sources coincide (e.g., different people use the same labels to name concrete things), but in other instances there may be discrepancies between sources, and the individual must weigh up each alternative to decide which is better or more correct. What criteria people use to make such decisions? Several studies indicate that individuals take into account different characteristics of the informants, both epistemic (e.g., knowledge attributed to the informant) and non-epistemic (e.g., physical attractiveness, group membership), depending on a variety of individual and situational factors.

Do the children follow any criterion to evaluate others’ testimony? To answer this question, researchers have used the experimental setting of the so-called conflicting-claims paradigm (Koenig et al., 2004), where participants are required to choose between conflicting information provided by two different sources. The usual findings indicate that preschoolers take into account some characteristics of the informants to selectively decide whom to trust (Harris et al., 2018). For example, children prefer to endorse information from an epistemic authority, such as a teacher, rather than from other types of informants (Corriveau and Harris, 2009), but they are also sensitive to the informant’s prior behavior. For instance, if the teacher previously provided blatantly wrong information (e.g., calling a spoon a duck) children tend to decrease their trust in him/her. In fact, the past accuracy of the source is among the most influential variables on children’s willingness to trust others’ testimony (Clément et al., 2004; Koenig and Harris, 2007; Pasquini et al., 2007), and from 7 years, they need just a single encounter with an inaccurate informant to use this information to make their trust decisions (Fitneva and Dunfield, 2010).

So far, in most of the studies with children, the informants were concrete persons making conflicting claims about some specific information. Surprisingly, there is very little research on the role of the Internet as a potential knowledge source, despite its undoubted significance across different settings in children’s lives (family, free time, school), and the public concern about the risk of fakes (e.g., Fun Kids, the United Kingdom’s radio station for children, includes a specific programe tackling fake news). Danovitch and Alzahabi (2013) examined how children evaluate technological informants, taking into account their prior history of accuracy. The results indicated that preschoolers considered this variable when deciding to trust (or not) the information provided by the device. However, this study did not explore children’s evaluation of the Internet as compared to a human source of information. To the best of our knowledge, only the recent research by Wang et al. (2019) has studied how children aged 5 to 8 years and adults evaluate information accessed on the Internet compared to that provided by a teacher. They found that for trivia-like questions (e.g., What color do you think Americans like best, yellow or purple?), older children more frequently endorsed the answer provided by a teacher, whereas younger children and adults showed no preference between the teacher and the Internet. However, for historical or scientific questions (e.g., How many days does it take Mars to complete a single orbit?), children of all ages showed no preference while adults chose the options provided by the Internet rather than those of a teacher. According to the authors, these apparent inconsistent patterns of trust choices may be due to a series of factors related to the exposure to the Internet and to the type of information itself. However, it is likely the experimental paradigm used in the study can also account for these findings.

Indeed, although the conflicting sources paradigm has yielded valuable information on specific aspects of children’s social learning, it is not exempt from criticism. One of the most noteworthy arguments has referred to the interpretation of the children’s responses when choosing between different sources of information (Lucas and Lewis, 2010). What does it mean, for example, when children choose informants who made correct judgments in previous trials rather than informants whose labeling was inexplicably wrong (calling a spoon a duck)? Some authors, under a rich interpretation, suggest this bias toward trust based on past accuracy reveals an ability to make epistemic judgments on what informants probably know (Koenig and Harris, 2007). Others, however, under a more parsimonious interpretation, contend that it involves relatively simple mechanisms in which epistemic inferences do not necessarily play a role (Lucas and Lewis, 2010). For example, children may reject inaccurate information because they believe the informant is not taking the task seriously, and this does not mean they attribute epistemic authority to the accurate informant. Nonetheless, despite the criticisms, the basic design of studies on trust in testimony remains useful for a first approach to under-studied topics.

In the present exploratory study, we use a basic conflicting source paradigm to explore the trust children and adults place in two sources of information with a great impact on everyday decisions: their teacher and the Internet. In addition, we examine how providing inaccurate information affects trust in each source. We selected primary school children, as they are wholly familiar with the use of the Internet. We chose 7 and 10-year-olds because previous studies have shown that from the age of 7 children are sensitive to inaccuracy based on just a single encounter, as we did here (Fitneva and Dunfield, 2010). We expected to find that children preferred their teacher to the Internet more than adults did, because during primary education, the teacher is a key figure of epistemic authority (Olson and Bruner, 1996). However, given there are virtually no previous studies comparing teachers and the Internet as sources of information, we made this prediction with some caution. Additionally, we expected the accuracy of the source to be related to participants’ subsequent trust, reducing their likelihood of endorsing the inaccurate source.

## Methods

### Participants

The participants were 57 second graders (29 females, *M*_age_ = 7.89 years, age range = 7.42–8.33 years), 57 fifth graders (28 females, *M*_age_ = 10.85 years, age range = 10.42–11.25 years), and 57 adults (41 females, *M*_age_ = 19.44 years, age range = 17.83–32.92 years) from the majority Spanish ethnic group. Children were recruited from a private school serving middle to upper-SES families in Toledo, Spain. Written parental consent, as well as children’s verbal assent, was obtained for all child participants. The adults were undergraduates in Education. Consent was also obtained from all adult participants.

### Procedure

The content presented in the study was prepared in collaboration with the school’s science teachers. We selected items that (1) were included in the primary education textbooks; (2) had not been presented and explained in class prior to the date of the experiment; and (3) were adequate to the children’s level of understanding and vocabulary. In total, 6 questions were presented in a booklet whose format was similar to those used in primary education class-time activities. For each question, two possible answers were provided: one by their real science teacher (at school for children, and at university for adults) with her photo below the answer, and another, obtained from an Internet website specialized in scientific information, with a photo of a computer showing the Google homepage on the screen.

The structure of the booklet was as follows: first, we presented two *baseline trials* (two questions whose answers were unlikely to be known, given the level of specificity and detail, e.g., How many eyes does a jellyfish have?), followed by the *intervention trial* (a familiar question to which the participants knew the answer, e.g., What do we call the sound made by dogs?), then we presented two *post-test trials* (two different unfamiliar questions), and finally, the *control trial* (a different familiar question) to ensure that the participants had maintained attention to the task. For the two familiar questions (intervention and control trials), one of the sources provided the accurate information (e.g., barking) and the other, inaccurate information (a pseudoword, e.g., cratting). In the intervention trial, half of the sample were presented with the teacher providing inaccurate information (*condition teacher-wrong*), while, for the remaining participants, the Internet was the inaccurate source (*condition Internet-wrong*). The reverse was true for the control trial. At the end of the booklet, there were some final questions on the participants’ gender and age.

For each condition, we counterbalanced (1) the position of the informant providing the correct answer (right-left) and (2) the source of the correct answer in the intervention trial (the Internet-teacher). This resulted in two different orders per condition.

The procedure was similar for all the participants. As part of a regular session at school or university, the researcher introduced herself and told the participants they were to complete the exercises in a booklet. The children were told that this would test their ideas about different scientific questions, but that their scores would not count toward their grades. The adult participants were told that the ultimate aim of the study was to prepare a primary education textbook and that the questions presented in the booklet had generated conflicting answers between the sources of information, their teacher and the Internet. The researcher insisted on the anonymous nature of the test, and that it would not be shown to anybody else at school or university. For the children, the testing was scheduled during science lessons, and to avoid any bias in their choices, their science teacher left the classroom beforehand. The researcher gave each participant their own booklet and presented the task collectively through the following example: “*When was the first bicycle made?” Your teacher [Name] told us that the first bicycle was manufactured in 1869 but we found on a specialized Internet website that the year was 1817*. *Now you have to mark with an X the answer you think is correct.* Once the researcher made sure all the participants understood the task, they were asked to open the booklet and individually mark the correct answer to each question. When all the participants had finished, they handed in the booklets.

### Data Analyses

Our dependent measure was the source of information selected by participants in each trial: the teacher or the Internet. We conducted analyses on group and individual performance for each age group. The former set of analyses^1^ addressed (1) whether the participants as a group chose one of the sources of information more often than chance in the two baseline trials as well as the two post-test trials (Binomial tests for each age group, with *n* = 114, *p* = 0.50); (2) to what extent the participants as a group changed their choices from baseline to post-test in each of the two conditions, teacher-wrong or Internet-wrong (McNemar tests); and (3) whether the accuracy of the source influenced the group performance by comparing participants’ choices during post-test in the two conditions (Chi-square tests). Additionally, we explored the age differences in the overall likelihood of choosing one of the two sources in any phase (Chi-square tests). The latter analyses focused on the individual trend of responses. We first explored the number of participants in each age group who consistently chose one of the sources in two of the two baseline trials and explored their choices after the intervention trial (i.e., inaccuracy of one of the sources). Due to the small and uneven number of participants displaying a preference for the teacher vs. the Internet, the analyses were conducted on the data in conjunction, regardless of the type of source preferred (teacher, Internet). Specifically, we ran Chi-square tests to investigate participants’ willingness to change (or maintain) their preference for a given source after experiencing their preferred source providing inaccurate information.

## Results

Preliminary analyses showed that the order of presentation did not affect the participants’ choices, Mann-Whitney *U*-tests: 7-year-olds: *U* = 400.00, 10-year-olds: *U* = 359.50, and adults: *U* = 304.00, *ps* > 0.05. Likewise, males and females did not differ in their general pattern of choices, Mann-Whitney *U*-tests: 7-year-olds: *U* = 382.00, 10-year-olds: *U* = 395.50, and adults: *U* = 253.00, *ps* > 0.05.

### Familiar Questions

Practically all the 7-year-olds (98.5%), the 10-year-olds (98.3%) and the adults (96.5%) correctly answered the *intervention trial*, regardless of the condition, teacher-wrong and Internet-wrong. Likewise, 96.9% of the 7-year-olds, 98.3% of the 10-year-olds, and 96.5% of adults correctly answered the last *control trial*. Crucially, no differences were found between the intervention and the control trials.

### Baseline Trials

**FIGURE 1:**
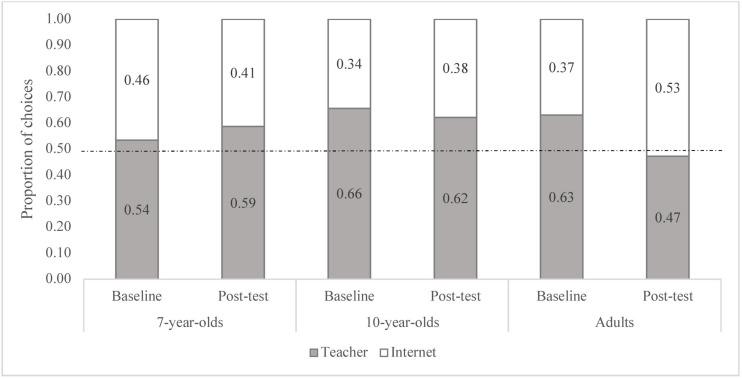
Proportion of responses siding with the teacher and the Internet for each age group during baseline and post-test phases.

The age differences in the frequency of choices during baseline were found to be non-significant [*X*^2^(2, *N* = 342) = 4.00, *p* = 0.135]. The analyses split by age show that the choices made by the 7-year-olds were not different from chance, Binomial test *p* = 0.512 (see Figure 1). Indeed, only 31.5% of the 7-year-olds consistently chose one of the two sources at baseline (*N* = 20, with 12 children choosing the teacher and 8 the Internet, in the two baseline trials). In contrast, for both the 10-year-olds and the adults, the most frequent choice was the teacher (10-year-olds: Binomial test *p* < 0.001, Cohen’s *g* = 0.16; Adults: Binomial test *p* = 0.006, Cohen’s *g* = 0.13). Regarding individual performance, we found that of the10-year-olds who consistently chose the same source (*N* = 22, 38.6%), the large majority preferred the teacher (20 of 22). Likewise, the adults displaying a preference for a given source (*N* = 31, 54.4%) usually preferred the teacher (23 of 31).

### Post-test Trials

As for the baseline, the 7-year-olds as a group did not systematically choose one of the two sources in the post-test trials (Binomial test *p* = 0.08), and the 10-year-olds continued to choose the teacher more often (Binomial test *p* = 0.011, Cohen’s *g* = 0.12). However, the adults’ responses during post-test were not different from chance level, in contrast to baseline (Binomial test *p* = 0.640) (see Figure 1). The age differences during post-test were marginally significant [χ*^2^*(2, *N* = 342) = 5.63, *p* = 0.06, Cramer’s *V* = 0.13], indicating, in line with this latter finding, that the adults tended to choose the teacher less often than did the 7- and 10-year-olds.

The analyses of the effect of the source accuracy show that the 7-year-olds and the adults did not change their overall choices from baseline to post-test in either of the two conditions (McNemar tests, *p* > 0.05). Moreover, in both age groups, the likelihood of choosing a given source in the teacher-wrong and the Internet-wrong conditions was similar [7-year-olds: *X*^2^(1, *N* = 114) = 0.06, *p* = 0.814; Adults: χ*^2^*(1, *N* = 114) = 0.33, *p* = 0.567]. A closer look at the participants who displayed a preference for a given source during baseline yielded a similar picture. For these particular subsamples (20 7-year-olds and 31 adults), participants’ choices after intervention were independent of whether or not their preferred source, either the teacher or the Internet, previously provided inaccurate information, Chi-square tests, 7-year-olds: *X*^2^(1, *N* = 20) = 0.01, *p* = 0.908; Adults: *X*^2^ (1, *N* = 31) = 0.004, *p* = 0.952 (see Table 1).

**TABLE 1:** Percentage (and number) of participants who chose the same source or switched the source immediately after intervention as a function of whether the source was accurate or inaccurate.

	**7-year-olds (*n* = 20)**		**10-year-olds (*n* = 22)**		**Adults (*n* = 31)**	
	**Accuracy**	**Inaccuracy**	**Accuracy**	**Inaccuracy**	**Accuracy**	**Inaccuracy**
Same choice	50 (10)	30 (6)	18.2 (4)	45.5 (10)	25.8 (8)	35.5 (11)
Switched choice	15 (3)	5 (1)	31.8 (7)	4.5 (1)	19.4 (6)	19.4 (6)

*Note that the subsamples for each age group corresponds to those participants who displayed a preference for a given source during baseline (i.e., choosing a source -either the teacher or the internet- in two of the two baseline trials).*

By contrast, the 10-year-olds overall made their choice depending on the condition in which they participated [*X*^2^(1, *N* = 114) = 5.60, *p* = 0.018, Cramer’s *V* = 0.22], but they did so in an unexpected way: Children in the teacher-wrong condition were more likely to choose the teacher whereas the children in the Internet-wrong condition chose the Internet more often. This finding was also confirmed by the analysis of the 10-year-olds who displayed a preference for a given source during baseline (*N* = 22). These participants were more likely to keep choosing the same source after their preferred source had provided inaccurate information compared to when (s)he/it was accurate, Chi-square test *X*^2^(1, *N* = 22) = 4.91, *p* = 0.027, Cramer’s *V* = *0.57* (see Table 1).

To sum up, the 7-year-olds did not systematically trust a particular source of information, and crucially, were impervious to the accuracy of the source when deciding what to choose. By contrast, the 10-year-olds displayed a preference for the teacher and seemed to be sensitive to the accuracy of the source, but they did so in a counter-intuitive way, as they preferred to trust the inaccurate source. Finally, the adults started by displaying a preference for the teacher but as the study progressed, they chose by chance, ignoring whether or not the source of information provided inaccurate information.

## Discussion

The main aim of this exploratory study was to examine the trust placed by children and adults in information provided by two sources, their teacher and the Internet, and the impact of the sources’ (in)accuracy on the participants’ willingness to trust. We designed a task based on the conflicting source paradigm, a frequently used format in research on trust in testimony. As in most previous works, we investigated the group performance, analyzing the most frequent choice in the samples, but we were also interested in looking at individual performance in order to obtain a deeper understanding of the phenomenon (Guerrero et al., 2017a; Juteau et al., 2019; Cossette et al., 2020). Our findings fail to confirm our two general hypotheses. First, children did not prefer their teacher to the Internet more than adults did. In fact, no consistent age differences were found in the participants’ willingness to choose either source. Second, accuracy did not have the expected influence either on the children’s choices or on those of the adults.

Regarding the baseline, whereas in the group analyses we found a *discreet* overall preference for the teacher over the Internet, the participants’ individual performance showed most participants alternated their choices between the teacher and the Internet, suggesting they did not display a consistent preference for a given source. Despite the unexpected nature of our findings, they do partially coincide with the heterogeneous results found by Wang et al. (2019). One possible explanation for the lack of source preference among most of our participants is that they tended to consider both sources (teacher and Internet) equally likely to be trustworthy. In this hypothetical case, their selections would be grounded in aspects other than the type of source. Unfortunately, as in other works using the basic conflicting sources paradigm, the study design does not provide information on the criteria participants leverage to make their choices. Nonetheless, studies in which participants were also asked the reasons for their choice of source may provide some clues (Guerrero et al., 2017a, b). In these works, most children unexpectedly failed to show a systematic preference for any source, not even in the presence of an epistemic authority such as a teacher. Children’s justifications suggested they focused on the piece of information, ignoring the source (e.g., “I think it’s a *reso* because it has *reso-like* things” -*reso* being a Spanish pseudoword applied to a new object). Whether or not this represents a common strategy when dealing with this type of paradigm should be addressed in future research.

What occurs after controlling for the accuracy of the sources is difficult to explain, at least from a rational, decision-making perspective. Overall, participants did not decrease their trust in a given source after providing inaccurate information. This is in contrast with numerous studies reporting that at around 4 years of age children present a preference for previously accurate vs. inaccurate sources (Clément et al., 2004; Koenig et al., 2004; Koenig and Harris, 2007), with one trial being sufficient (Pasquini et al., 2007), from the age of 7, for them to discriminate between accurate and inaccurate informants (Fitneva and Dunfield, 2010). This unexpected finding leads to the proposal of two not mutually exclusive explanations: one related to participants’ evaluation of the informants’ inaccuracy, and the other related to the paradigm itself.

The former explanation is related, in line with previous research, to individuals not always considering past accuracy as an absolute epistemic condition (Einav and Robinson, 2011; Kushnir and Koenig, 2017). In other words, an informant’ making a mistake does not mean (s)he automatically loses credibility. Children -and adults- may excuse inaccuracies for different reasons (Nurmsoo and Robinson, 2009; Kondrad and Jaswal, 2012), or even interpret them as jokes or a fun way to answer, obviating the epistemic context (e.g., Henderson et al., 2015). In the present study, the inaccuracies are incorrect claims (e.g., “cratting” instead of barking), but in contrast to previous works, as underlined by Lucas and Lewis (2010, p. 168), they are not “incorrect in a manner that cannot be made sense of” (e.g., calling a spoon a duck). Although most of the participants chose the familiar term in the intervention trial, the alternative label could have been interpreted as an unconventional but plausible term. This would explain why the inaccuracies failed to condition the participants’ subsequent responses in the expected way. Other works using the conflicting sources paradigm but presenting plausible inaccuracies report similar findings. Guerrero et al. (2017a) found that when a teacher (vs. an unfamiliar informant) proposes a non-conventional but plausible use for a familiar object (e.g., using a toy bucket to serve a salad), the pre-schoolers’ trust in the source remains unaffected. Arguably, as proposed by Lascaux (2020), the variety of strategies that individuals may use in these tasks does not always yield epistemic benefits, but other type of benefits, either social (e.g., group cohesion when trusting dominant in-group informants) or cognitive (diminishing the inconsistencies between what an individual expects from the informant and what really happens). Unfortunately, the paradigm used here does not allow to know whether or not individuals take an epistemic glance at the task, which is crucial in the study of testimonial learning.

This is directly related to the second explanation we propose, namely, the validity of the conflicting sources paradigm. The vast majority of the studies using this paradigm conclude that children, from a very early age, pay attention to the informant’s past accuracy, their accent, physical attractiveness, familiarity, social status, etc. (Harris et al., 2018). However, recent published studies report findings that are inconclusive or contrary to expectations, calling the validity of the paradigm into question. Recently, Juteau et al. (2019) used this paradigm to test whether children displayed a consistent preference to learn from a confident informant (vs. a non-confident informant). They found a substantial lack of consistency between the same participant’s responses in similar tasks with different content, or even in the same task performed at different times. More related to our goals, Cossette et al. (2020) explored how accuracy influenced children’s trust choices, finding that although the group tended to side with the previously accurate informant, the stability of participants’ choices was surprisingly disrupted by superficial aspects of the task (e.g., order of presentation) or even by extraneous variables (e.g., their particular mood or a desire to be “silly” at a given moment). Along with the present findings, these studies highlight that the format itself of the conflicting sources paradigm fails to capture the underlying reasons for participants’ choices, which largely depend on the individual’s interpretation (see Lucas and Lewis, 2010, for a comprehensive criticism of the paradigm).

To sum up, future research needs to broaden its horizons to fully understand how individuals selectively trust others. On the one hand, as regards the treatment of the data obtained using the paradigm, we consider it essential to analyze both individual and group performance. In this regard, it would be interesting to perform a meta-analysis of the studies that, to date, have explored children’s selective trust using the conflicting sources paradigm from the perspective of individual differences (see Tong et al., 2019, for a meta-analysis of group tendencies). This proposed analysis would allow to determine to what extent conclusions based on group data are confirmed at individual level. On the other hand, it is necessary to study learning by testimony using an approach with more realistic experimental designs, which allows researchers to explore the participants’ underlying motives for their choices. This will ultimately contribute to the understanding of the foundations of selective social learning throughout childhood and beyond.

## Data Availability Statement

The raw data supporting the conclusions of this article will be made available by the authors, without undue reservation, to any qualified researcher.

## Ethics Statement

The studies involving human participants were reviewed and approved by the UCLM. Written informed consent to participate in this study was provided by the participants’ legal guardian/next of kin.

## Author Contributions

SG and IE conceived the project and designed the study. CS-E also contributed to the study design and analyzed the data. EV and IM collected the data and also helped to the data analyses. SG, CS-E, and IE wrote the manuscript. All authors contributed to the article and approved the submitted version.

## Conflict of Interest

The authors declare that the research was conducted in the absence of any commercial or financial relationships that could be construed as a potential conflict of interest.

## Footnotes

1 Following the statistical approach of prior studies using this paradigm, the analyses on the group performance were conducted on the participants’ choices in the two trials of each phase, baseline and post-test. Thus, the total sample for each age group corresponded to *n* = 114. Given that this represents a violation of the independence assumption, results should be interpreted cautiously.
